# Endocytosis at the maternal-fetal interface: balancing nutrient transport and pathogen defense

**DOI:** 10.3389/fimmu.2024.1415794

**Published:** 2024-06-18

**Authors:** Mingming Fan, Hongyu Wu, Amanda N. Sferruzzi-Perri, Yan-Ling Wang, Xuan Shao

**Affiliations:** ^1^ State Key Laboratory of Stem Cell and Reproductive Biology, Institute of Zoology, Chinese Academy of Sciences, Beijing, China; ^2^ Key Laboratory of Organ Regeneration and Reconstruction, Institute of Zoology, Chinese Academy of Sciences, Beijing, China; ^3^ University of Chinese Academy of Sciences, Beijing, China; ^4^ Centre for Trophoblast Research, Department of Physiology, Development and Neuroscience, University of Cambridge, Cambridge, United Kingdom; ^5^ Beijing Institute for Stem Cell and Regenerative Medicine, Beijing, China; ^6^ Institute for Stem Cell and Regeneration, Chinese Academy of Sciences, Beijing, China

**Keywords:** endocytosis, placenta, decidua, macropinocytosis, nutrient, pathogen

## Abstract

Endocytosis represents a category of regulated active transport mechanisms. These encompass clathrin-dependent and -independent mechanisms, as well as fluid phase micropinocytosis and macropinocytosis, each demonstrating varying degrees of specificity and capacity. Collectively, these mechanisms facilitate the internalization of cargo into cellular vesicles. Pregnancy is one such physiological state during which endocytosis may play critical roles. A successful pregnancy necessitates ongoing communication between maternal and fetal cells at the maternal-fetal interface to ensure immunologic tolerance for the semi-allogenic fetus whilst providing adequate protection against infection from pathogens, such as viruses and bacteria. It also requires transport of nutrients across the maternal-fetal interface, but restriction of potentially harmful chemicals and drugs to allow fetal development. In this context, trogocytosis, a specific form of endocytosis, plays a crucial role in immunological tolerance and infection prevention. Endocytosis is also thought to play a significant role in nutrient and toxin handling at the maternal-fetal interface, though its mechanisms remain less understood. A comprehensive understanding of endocytosis and its mechanisms not only enhances our knowledge of maternal-fetal interactions but is also essential for identifying the pathogenesis of pregnancy pathologies and providing new avenues for therapeutic intervention.

## 1 Introduction

A successful pregnancy necessitates a continuous dialogue between the mother and developing conceptus. This communication is facilitated by intricate interactions between maternal and fetal cells at specific sites in the uterus, collectively referred to as the “maternal-fetal interface.” The placenta plays an instrumental role in fetal growth and health by enabling nutrient uptake from the mother’s blood, facilitating waste removal from the fetus’s blood, ensuring immunological tolerance for the semi-allogenic fetus, and acting as a protective barrier against toxins and pathogens (1–3). The distribution of nutrients between the mother and fetus during pregnancy is predominantly influenced by these maternal-fetal interactions, which are largely regulated by the placenta. Nutrient transport across the maternal-fetal interface occurs through various pathways: passive transport, facilitated diffusion, active transport, and endocytosis (4). While the mechanisms of placental transfer via passive diffusion or membrane transporters have been extensively studied, the roles of alternative mechanisms remain less understood (4). This review aims to provide a comprehensive understanding of endocytosis at the maternal-fetal interface, with a particular focus on the placenta, to elucidate the role of endocytosis in balancing nutrient transfer and pathogen defense.

## 2 Functional units at the maternal-fetal interfaces in human pregnancy

**Figure 1:**
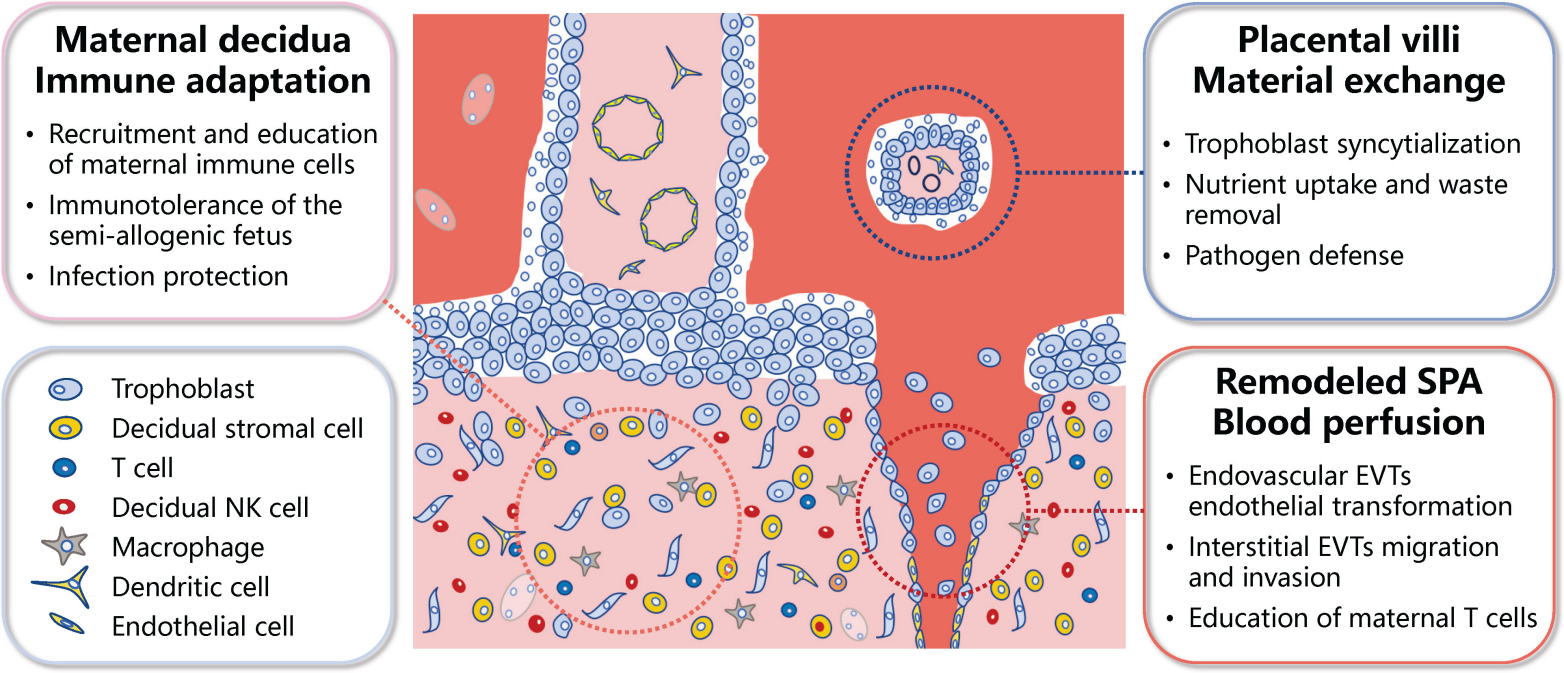
Schematic representation of the primary functional units of the human placenta. The meticulous differentiation of placental trophoblasts, coupled with the dynamic interplay between these trophoblasts and a myriad of maternal cells at the maternal-fetal interface, culminates in several functional units within the placenta. These primarily encompass the immune adaptation unit at the maternal-fetal interface, the maternal-fetal material exchange unit, and unit of the blood perfusion into the maternal-fetal interface. SPA, spiral artery; EVTs, extravillous trophoblasts; NK, natural killer.

The well-organized differentiation of trophoblasts and the complex interactions among trophoblasts and a multitude of maternal cells at the maternal-fetal interface results in the formation of several functional units. The first unit involves the uterine decidua, where the interactions among decidual stromal cells, maternal immune cells, and trophoblast cells of fetal origin ensure immune tolerance to the semi-allogenic fetus, while protect against placental/fetal infections (2, 3, 5). The second functional unit is the placental villi, which are fundamental for maternal-fetal material exchange. The multinucleated syncytiotrophoblast (STB), which lines the outer surface of the placental villi, is formed through the fusion of mononucleated cytotrophoblast (CTB) cells. The STB serves as the primary site for maternal-fetal nutrient transport and waste removal, while also acts as a barrier to prevent pathogen infection (2, 3, 6). The third functional unit is the transformed uterine spiral arteries, which enable the perfusion of maternal blood into the maternal-fetal interface. Interactions between extravillous trophoblasts (EVTs; interstitial and endovascular), maternal immune cells, and vascular smooth muscle cells are needed for the transformation/remodeling of the uterine spiral arteries into high-capacity, low-resistance arteries (2, 3, 7) (**Figure 1**).

### 2.1 Maternal decidua—functional unit of immune adaptation at the maternal-fetal interface

One main placental function balancing sustained immunologic tolerance to the semi-allogenic fetus while providing adequate protective immunity against infection, which have been interpreted as the “paradox of pregnancy” (8). During early pregnancy, maternal immune cells populate the uterine mucosa, and primarily comprise decidual natural killer (dNK) cells (approximately 50%–70%), followed by macrophages (around 20%), and T cells (between 10%–20%), with dendritic cells (DC), mast cells, and B cells having a minor presence (5, 8). Notably, dNK cells, characterized by their low cytotoxicity and robust cytokine-producing capacity, perform multiple functions related to immune tolerance, embryo development, and protection against infection. EVTs express human leukocyte antigen-C (HLA-C), HLA-G, and HLA-E (9–11). These are recognized by inhibitory receptors on dNK cells, thereby reducing cytotoxicity and enhancing the secretion capability of dNK cells, contributing to the regulation of trophoblast invasion and differentiation, remodeling of uterine spiral arteries, and lymphatic mimicry (12–14). The interaction of immune checkpoint inhibitor programmed death-1 (PD-1) and its ligand programmed death ligand-1 (PD-L1) also plays a significant role in establishing immune tolerance at the maternal-fetal interface (2). PD-L1 is predominantly expressed by EVTs and villous STB (15) and its recognition through interacting with PD-1 on decidual immune cells, including macrophages and CD8^+^ T cells, limits an immune response from being activated. It could also contribute to the formation of M2 polarized macrophages and inhibit the cytotoxicity of CD8^+^ T cells (16). Decidual macrophages function to clear apoptotic trophoblasts through their phagocytic activity (8). They also produce various factors, including vascular endothelial growth factor (VEGF) and matrix metalloproteinase-9 (MMP9), which promote angiogenesis and tissue remodeling (8). Decidual macrophages also regulate both adaptive T cell responses and innate NK cell response (17).

### 2.2 Placental villi—functional unit of maternal-fetal material exchange

The syncytium, the outermost layer of the placental villi, is a continuous surface that is estimated to extend 12 to 14 m^2^ at term. It is directly exposed to maternal blood and thus ideally poised to act as the primary site for regulating maternal-fetal gas, nutrient, and waste exchange, whilst serving as the frontline for pathogen defense (2, 3, 6). The STB possesses a range of transporters for glucose, amino acids, and fatty acids, indicating their preferential uptake of these low molecular-weight nutrients (6). Most amino acids are transported from the maternal circulation into the fetal circulation via active transport systems across the placenta. Of particular interest are the Na^+^- dependent and -independent amino acid transporters, which have been identified in both the apical (maternal) and basal (fetal) facing membranes of the STB of the human placenta (18–20). Total transport rate of a specific amino acid is influenced by its concentration in the maternal versus fetal circulation, and placental surface area, abundance, affinity, and activity of amino acid transporters (21–23). In addition to transporters, recent research has revealed that macropinocytosis, a fluid-phase endocytosis characterized by the formation of large vesicles at the sites of membrane ruffling, functions in the STB to modulate amino acid uptake (6). Although the STB also functions as a barrier to pathogens, some pathogens can pass through STB and infect the fetus. These include Zika virus (ZIKV) and severe acute respiratory syndrome coronavirus 2 (SARS-CoV-2) (24–29).

### 2.3 Remodeled uterine spiral arteries—functional unit of blood perfusion into the maternal-fetal interface

In placental mammals, the process of invasion by EVTs into the decidua and the subsequent remodeling of uterine spiral arteries is most prominently observed in humans (30). This phenomenon is postulated to ensure an ample supply of nutrients and oxygen for the intrauterine development of the large human brain (31). The enEVTs play a pivotal role in this process as they invade the spiral arteries, eliminate the smooth muscle cells, and replace the endothelial cells (32–35). Additionally, iEVTs are instrumental as they infiltrate the decidual stroma and contribute to the depletion of vascular smooth muscle cells (36, 37). Following remodeling, the spiral arteries display lymphatic markers such as PROX1 (prospero homeobox gene 1), LYVE1 (lymphatic vessel endothelial hyaluronan receptor 1), and VEGFR3 (vascular endothelial growth factor C (VEGF-C)/VEGF receptor 3) (12). This is partly dependent on dNK cells, which secrete VEGFC and is needed for the activation of VEGFR3 and remodeling of spiral arteries (12). Recent studies have also highlighted the immune regulatory properties of enEVTs. These cells produce transforming growth factor-β1 (TGF-β1) and induce the differentiation of maternal Treg which perform a central role in shielding the fetus from maternal immune attack (7). Transcriptomic analysis also suggests enEVTs have a gene expression profile particularly mirroring that of M2 macrophages and produce various cytokines and chemokines (31).

## 3 Receptor-mediated endocytosis at the maternal-fetal interface

Receptor-mediated endocytosis (RME) is the most comprehensively characterized pathway for macromolecules and macromolecular complexes (ligands) to enter cells. Upon binding to their respective transmembrane receptors, ligands are internalized into endocytic vesicles that fuse to form early endosomes (38, 39). The sorting process of these internalized ligand-receptor complexes depends on their type; metabolic receptors are recycled back to the plasma membrane, whereas signaling receptors and their associated ligands (e.g. receptor tyrosine kinases or receptors coupled with tyrosine kinase) are delivered to the internal vesicles of multivesicular late endosomes, which are degraded after interaction with lysosomes. During this process, endosomes move from the periphery of the cell to the juxtanuclear region, where they undergo multiple fusion, invagination, tabulation, and membrane fission events (38, 39). Clathrin-mediated endocytosis (CME) is the most extensively studied RME, although clathrin independent endocytosis (CIE) also plays a significant role. This section will provide a comprehensive review of the advancements in research concerning these two endocytosis pathways at the maternal-fetal interface.

### 3.1 Clathrin-mediated endocytosis

#### 3.1.1 Brief introduction

CME is a receptor-mediated endocytosis during which cargo is packaged into vesicles with the assistance of a clathrin coat. The complexity of the clathrin-mediated endocytic pathway is illustrated by the increasing number of alternative cofactors, adaptors, and tethering proteins that are involved in the formation of clathrin-coated pits and vesicles (40). The process involves several stages, including initiation, stabilization, maturation, fission, and uncoating (41). Initially, cargo is selected and bound by coat-associated clathrin-adaptor proteins, such as the AP2 complex and scaffold proteins. Clathrin triskelia are recruited directly from the cytosol to the adaptor region of the plasma membrane. Subsequently, the membrane bends, transforming the flat plasma membrane into a “clathrin-coated pit” (CCP), where clathrin polymerizes in hexagons and pentagons around a developing vesicle bud, thereby stabilizing membrane curvature. Following this, GTPase dynamin is recruited to the neck of the CCP by BAR domain-containing proteins and catalyzes the scission of the vesicle from the plasma membrane, releasing the CCP as a clathrin-coated vesicle (42). When clathrin-coated vesicle scission occurs, a gap in the clathrin cage forms, which facilitates the uncoating apparatus, including auxilin and HSC70, to initiate the process of removing the clathrin coat. Ultimately, the disassembly of the clathrin coat releases the nascent cargo-filled vesicle, allowing it to be trafficked further within the cell or transported out of the cell via transcytosis (42, 43). The clathrin machinery is returned back into the cytoplasm to be enlisted and reused for another cycle of clathrin-coated vesicle formation. Depending on cargo versatility, CME has various functions, including nutrient transport into cells, sampling of the cell’s environment for growth and guidance cues, and the control of signaling pathway activation (42). Notably, pathogens, including certain viruses like SARS-CoV-2 and ZIKV can also enter cells facilitated by this process (42).

#### 3.1.2 CME functions as a mechanism for nutrition and drug delivery at the maternal-fetal interface

Transmission electron microscopy has revealed that CME occurs within the placental STB, with coated vesicles observed at the microvillous membrane of the STB (44). Transferrin-bound iron and albumin, which are well-established cargo in the placental STB, are transported via CME (45–47). The transferrin-bound iron is internalized by transferrin receptor 1 (TfR1) at the microvillous membrane of the STB (48). Within endosomes, iron then separates from transferrin, undergoes reduction, and then is released into the cytoplasm (49). Albumin, a serum protein, binds a variety of cargos in the blood, including vitamin D and fatty acids (50). Albumin complexes can be transported to the placenta by megalin and cubilin, which are expressed in placental STB (45) and increase during gestation (51). These data suggest that megalin and cubilin may play a significant role in maternal-fetal transfer.

Megalin is a 600-kDa multi-ligand receptor of the low-density lipoprotein receptor-related protein family, characterized by a large amino-terminal extracellular domain, a single transmembrane domain and a short carboxy-terminal cytoplasmic tail believed to have signaling functions (52). Cubilin is a 460-kDa peripheral membrane glycoprotein, with an initial amino-terminal of 110 amino acids, eight epidermal growth factor (EGF)-like repeats and 27 CUB (complement subcomponents C1r/C1s, EGF-related sea urchin protein and bone morphogenic protein-1) domains (53). Cubilin lacks the transmembrane intracellular domain and always works in conjunction with megalin to perform its endocytic function (54). These two multi-ligand receptors have been extensively studied within the kidneys, where they are prominently expressed in the apical region of epithelial cells in the proximal tubule and co-localized with clathrin coated pits and endosomes (55). As previously reported, their potential ligands encompass vitamin B12 and D, folic acid, retinoic acid, albumin, transferrin, apolipoproteins, cholesterol, insulin, EGF, calcium, and aminoglycosides (56). It is postulated that megalin and cubilin may similarly participate in the transport of these cargo by the placenta. However, the molecular mechanisms governing megalin and/or cubilin-mediated transplacental transport of most substrates remain uncharacterized at the maternal-fetal interface.

Furthermore, megalin plays a role in the placental transport of aminoglycoside antibiotics, including gentamicin, used for treating of intra-amniotic infections (57). Consequently, megalin holds potential as a ligand-modified drug nanocarriers designed to deliver therapeutic molecules to the placenta. Of note, PEGylated lipids modified with gentamicin have been shown to undergo clathrin-mediated uptake that involve megalin (58). Furthermore, megalin is needed for the uptake of a liposome encapsulated fluorescence probe in BeWo cells (a choriocarcinoma cell line) (57).

#### 3.1.3 CME can facilitate the entry of viruses at the maternal-fetal interface

In addition to its role in nutrient transport and drug delivery, CME can also serve as a mechanism for the entry of specific viruses into the placenta. SARS-CoV-2, a novel coronavirus responsible for the severe disease known as Coronavirus Disease 2019 (COVID-19), has spread to over 200 countries globally. A multitude of studies have identified the presence of SARS-CoV-2 virus in placental tissue from infected pregnant women, suggesting that villous STB may be the primary target of infection (24–27). The entry factors for SARS-CoV-2 infection, angiotensin-converting enzyme 2 (ACE2) and transmembrane serine protease 2 (TMPRSS2) (59), are also expressed by the human placenta (60, 61). Nevertheless, infection can still occur if TMPRSS2 is inadequate, during which the virus–ACE2 complex is internalized via CME (62).

ZIKV has the potential to vertically transmit from an infected mother to her fetus, thereby significantly impacting fetal health. This can lead to conditions such as microcephaly, congenital malformations, and fetal demise (28, 29). Studies conducted using the choriocarcinoma cell lines JEG-3 have shown that ZIKV crosses the placental barrier via both the paracellular pathway and the endocytic and transcytotic pathways (63). In the paracellular pathway, ZIKV infection can disrupt tight junctions, thereby allowing the virus to traverse the compromised barrier. As shown by the use of inhibitors, the endocytic and transcytotic pathways mediating viral entry are facilitated by CME, caveolae-dependent endocytosis and macropinocytosis (63). However, there is a need to understand which specific cell surface receptor(s) are involved in this process. Efforts employing single-cell RNA-sequencing and immunohistochemistry have identified AXL as a potential ZIKV entry receptor in neural stem cells (64). Despite this, knockout of AXL did not protect human neural progenitor cells and cerebral organoids from ZIKV infection (65). However, recent research using gene knockout and overexpression strategies in human trophoblast stem cells and organoids has shown that AXL and TIM-1, another putative ZIKV entry receptor contribute to the high sensitivity of the placenta to ZIKV infection (66). Therefore, AXL and TIM-1 likely work together to enable ZIKV infection at the maternal-fetal interface, but further research is required.

#### 3.1.4 CME can serve as a bridge for intercellular communication at the maternal-fetal interface

CME is crucial for the communication between the mother and fetus. As previously mentioned, the largest population of leukocytes at the maternal-fetal interface are dNK cells, which exhibit low cytotoxicity. dNK cells interact with HLA-G^+^ EVTs as the EVTs invade the maternal decidua. Prior studies have shown that upon interaction with EVTs, dNKs acquire HLA-G via trogocytosis, a specific form of endocytosis (67). Mechanistically, filamentous projections from HLA-G-enriched EVTs establish synapses with dNK cells, facilitating direct HLA-G transfer (67). The HLA-G then interacts with dNK cell expressed KIR2DL4, forming a complex that undergoes GTP-dependent internalization via dynamin-mediated CME (68). Data suggests that intracellular HLA-G–KIR2DL4 complexes can lead to sustained intracellular signaling following the abrogation of the immune synapse and uptake of HLA-G. Cytokine activation promotes HLA-G degradation and coincides with increased cytotoxicity by dNK cells. This uptake of EVT HLA-G by trogocytosis confers an immuno-suppressive characteristic to dNK cells, reducing their cytotoxicity. Notably, the disruption of this process by viral products leads to activation of cytotoxicity of dNK cells and the control over virus infection at the maternal-fetal interface (67). Thus, this process provides both maternal-fetal immune tolerance and anti-viral immune function by dNK cells (67).

Maternal Treg cells are pivotal in the immune tolerance of the semi-allograft fetus. These CD4^+^CD25^+^Foxp3^+^ Tregs constitutively and significantly express the immune checkpoint receptor cytotoxic T-lymphocyte antigen 4 (CTLA-4) (69). The protective effect of Tregs on the fetus involve CTLA-4 and the formation of immune synapses on these Treg cells. These interactions facilitate the depletion of CD80/CD86 costimulatory molecules on antigen presenting cells (APCs) through CTLA-4-dependent trogocytosis (69). Furthermore, the downregulation of CD80 by Treg-expressed membrane CTLA-4 disrupts cis-CD80/PD-L1 heterodimers on DCs. This disruption results in the release of soluble PD-L1, which inhibits the activation of PD-1-expressing effector T cells (69). Consequently, this ensures immunotolerance towards the semi-allograft fetus.

Other studies have revealed that the placenta is able to internalize macrophage-derived exosomes via CME, which subsequently modulates the production of cytokines by the placenta, including interleukin (IL)-6, IL-8, IL-10, and IL-12 (70). This mechanism by which immune cells can signal to the placental unit may aid responses to maternal inflammation and infection and thereby, serve to limit harm to the fetus. Indeed, researchers have highlighted that potential role of macrophage-derived exosomes as therapeutics for clinical translation (71). However, further work is needed to study their benefit at the maternal-fetal interface and in improving pregnancy outcomes.

### 3.2 Clathrin independent endocytosis

#### 3.2.1 Brief introduction

While the CIE plays a crucial role in the cellular communication between cells with their surrounding environment, its understanding lags significantly behind that of the CME. The known functions of CIE encompass extracellular lipid/protein/virus uptake and removal of activated receptors from the plasma membrane to regulate cell polarization, spreading, and migration (72). Importantly, CIE regulates the cellular entry of over twenty viruses (including HIV, Herpes, Ebola, SV40 and Dengue viruses), various toxins, bacteria and prions (such as cholera and Shiga toxins), Streptolysin O and VacA (73). Furthermore, CIE has been identified in numerous *in vitro* cell lines and *in vivo* in mouse, worm, fly, plant, and yeast species (74). However, the mechanisms of CIE remain poorly characterized, particularly at the maternal-fetal interface.

CIE encompasses various types, including caveolin-mediated endocytosis, clathrin-independent carrier (CLIC)/glycosylphosphatidyl­inositol-anchored protein enriched early endocytic compartment (GEEC) endocytosis (both clathrin and dynamin independent), fast endophilin-mediated endocytosis (FEME, a clathrin-independent but dynamin-dependent pathway for rapid ligand-driven endocytosis of specific membrane proteins), and phagocytosis (75). Although caveolae can bud from the plasma membrane, few cargos rely on them for uptake. Due to the absence of specific endocytosis inhibitors, it is currently not possible to fully distinguish between the FEME or CLIC/GEEC pathways and the caveolae pathway.

#### 3.2.2 Caveolin-mediated endocytosis at the maternal-fetal interface

Caveolae-mediated endocytosis is characterized by their flask-shaped invagination of the plasma membrane. These structures are distinguished by their high concentrations of membrane cholesterol, sphingolipids, and integral membrane caveolin proteins. These components contribute to the curvature of the plasma membrane (76). The ATPase Eps15 homology (EH) domain-containing protein 2 (EHD2), a membrane remodeling protein, has been suggested to form a ring-like oligomer that encircles the caveolar neck. This protein plays a crucial role in stabilizing caveolae at the plasma membrane and regulating endocytosis (77). Following membrane invagination, caveolin-rich vesicles are extracted from the membrane via dynamin action (76). A wealth of literature has established a significant correlation between caveolae and endocytosis. This includes studies in vascular endothelia, suggesting that caveolae facilitate transcellular transport from the vessel lumen into tissues (78). Furthermore, beyond their role in endothelial cells, caveolae also play a comparable function in numerous endocytic processes, such as the uptake of toxins, viruses, entire bacteria, lipids, and various nanoparticles (75).

Caveolin-1 is expressed in the CTB of term placentas and its expression has also been observed in endothelial cells, pericytes and stromal cells of fetal capillaries but not in STB (79). Thus, caveolin-1-mediated endocytosis may be involved in nutrient uptake by the fetus. In a recent study, intracellular accumulation of caveolin-1 was observed with colocalization of Met in trophoblast isolated from early-onset preeclamptic placentas (80). Additionally, prolonged hypoxic stress was found to enhance endocytosis and inhibit ubiquitin-mediated Met degradation, resulting in impaired regulation of trophoblast invasion by hepatocyte growth factor (HGF)/Met signaling (80).

Riboflavin (vitamin B2) is involved in essential cellular redox reactions. In addition to CME, riboflavin may be taken up into the placenta via other endocytosis pathways. Studies in BeWo cells have demonstrated that riboflavin is co-localized with both clathrin- and Rab5-positive endosomes (81). Furthermore, the uptake of riboflavin required GTPase dynamin activity, suggesting an endocytic uptake mechanism (81). Colocalization of fluorescent ligand endocytosis assays with rhodamine-riboflavin and immuno-stained caveolin-1 in the BeWo cell line has suggested that the uptake of riboflavin involves multiple distinct endocytosis pathways (81). However, these results require confirmation in primary trophoblast or placental villous tissue. Riboflavin transport via specific carrier-mediated mechanisms has also been identified, with the human riboflavin transporter (hRFT1) being observed to be expressed by the human placenta (82). hRFT1 is typically found at the basal membrane of polarized epithelia (83, 84) and therefore, in the placenta, may facilitate riboflavin transport across the basal membrane from the STB to fetal blood. However, this has yet to be studied.

Endocytosis inhibitors, including those that affect caveolae, can block ZIKV transcytosis, suggesting that caveolae may also contribute to ZIKV transcytosis (63). Moreover, the most studied viruses that use caveolar/raft-dependent pathways belong to the polyomavirus family, which includes SV40, mouse polyomavirus (mPy), and two human pathogens, namely BK and JC viruses (85). Each of these viruses use different gangliosides as receptors (86–88). For instance, SV40 requires ganglioside GM1 for its binding, internalization and infection (89), whereas recent studies on mPy have shown that the receptor ganglioside GD1α is not required for binding and endocytosis and instead guides the virus from late endosomes to early endosomes (90). Although previous studies based on some observations have suggested that the uptake of SV40 into different host cells was associated with caveolae, subsequent *in vitro* cell line experiments and studies using mouse primary cells showed that most surface-bound SV40 and mPy in various cell types were not associated with plasma membrane caveolae (90–100). However, the fraction of virus that uses caveolar entry might differ between cell types and the virus; this is supported by the observation that when caveolin-1 was expressed in caveolin-deficient Jurkat cells, infection rates by mPy increased significantly (99). These results suggest that a small percentage of SV40, mPy and possibly other polyomaviruses enter the cytoplasm via the caveolin pathway (101).

While caveolin-mediated endocytosis is crucial for nutrient transport and pathogen entry at the maternal-fetal interface, the significance of caveolae-facilitated endocytosis remains largely unexplored. Further research is required to fully elucidate this matter.

#### 3.2.3 Other endocytosis pathways at the maternal-fetal interface

In addition to the previously mentioned caveolin-mediated endocytosis, numerous other CIE pathways exist. The nutritional demand for folate (vitamin B9) escalates to meet the developmental needs of the fetus during pregnancy. Folate uptake primarily occurs through glycosylphosphatidylinositol (GPI)-modified receptors and folate receptors, which are predominantly expressed in the microvillous membrane of the STB, and involve the CLIC/GEEC pathway (102, 103). Following uptake of folate by the STB, it is transported to the fetus via the STB basal membrane, a process potentially facilitated by the multi-drug resistance protein 1 (MRP1) (102). Endocytosis may also be involved in this process, as the endocytosis inhibitor monensin inhibited uptake of folate by the BeWo cell line (104). The CLIC/GEEC pathway also aids in the endocytosis of GPI-anchored proteins within uncoated tubulovesicular membrane structures, thereby contributing to fluid uptake (76). However, direct evidence for the uptake of cargo by STB through CLIC/GEEC pathway remains to be further investigated.

Transfer of maternal IgG into the fetal circulation confers passive immunity to the fetus. Prior research has established that in human intestinal epithelial cells, IgG is internalized, at least partially, through hFcRn-mediated endocytosis; mechanisms that are both clathrin- and caveolin-independent (105). Studies have shown that hFcRn plays a significant role in the transport of IgG via ex vivo perfused placenta (106). Moreover, it has been verified that IgG exhibits a high affinity for hFcRn within the acidic pH environment of endosomes, while IgG fails to bind to the cell surface under neutral pH conditions (106). Additionally, research in the BeWo cell line suggests that IgG is internalized via fluid-phase endocytosis, with the tight binding of IgG to hFcRn (107). Notably, hFcRn has been identified in the fetal capillary endothelium and STB of human placenta (108). The Fc gamma receptor IIb (FCGR2B) is also expressed by the villous fetal endothelium, however the mechanism underlying the uptake of IgG by the STB and subsequent transport of IgG across the fetal capillary endothelium remains incompletely understood (106, 108). Furthermore, the hFcRn receptor may play a role in the transplacental transport of human cytomegalovirus, which can lead to severe disabilities (4).

Flotillins, a category of integral membrane proteins linked with lipid microdomains, have been implicated in endocytosis processes independent of both clathrin and caveolae. Studies have indicated that flotillins, FLOT1 and FLOT2 are highly expressed in term villous CTBs and endothelial cells, while their expression in the STB is comparatively less (4, 109). This implies that flotillin-dependent endocytosis may not be the primary pathway in the STB, but could play functional roles in the CTB and endothelium. Additionally, flotillins are essential for the endocytosis of molecules including membrane-bound receptors like CD59, which is implicated in the control of complement activation (4, 109). Further investigations in the human placenta are therefore strongly recommended.

The FEME pathway, a recently identified critical route for the rapid endocytosis of specific transmembrane receptors, is characterized by its independence from clathrin and dependence on dynamin. This pathway plays a pivotal role in a renal cell line (BSC1; a cell line isolated from the kidney of an African green monkey) migration and growth factor signaling (110). Despite these findings, the significance of the FEME pathway at the maternal-fetal interface remains ambiguous and warrants further exploration.

Studies of RME at the maternal-fetal interface are both extensive and challenging. However, as summarized above, current evidence indicates that RME may be critical for facilitating the transfer of various nutrients from the maternal blood to the fetal circulation, whilst also by providing a route for drug and pathogen entry into trophoblast cells.

## 4 Fluid-phase endocytosis

### 4.1 Brief introduction of macropinocytosis

Although the mechanisms described in the preceding section can be described as RME, which is primarily responsible for internalizing solid substances, pinocytosis describes a form of fluid-phase endocytosis. Fluid-phase uptake in cells occurs through two distinct processes, namely micropinocytosis and macropinocytosis. Micropinocytosis is a non-specific process that results in small vesicles of less than 0.1 μm in diameter and involves both clathrin-coated and non-coated vesicles (111, 112). Meanwhile, macropinocytosis, a specialized process exclusive to certain cell types, involves the formation of large vesicles measuring between 0.2–5 μm in diameter that form at sites of membrane ruffling (111, 113). Macropinocytic vacuoles (macropinosomes) are formed when membrane ruffles fold back onto the plasma membrane to form fluid-filled cavities that close by membrane fusion. Macropinosome formation is not guided by a particle or a cytoplasmic coat, and this gives rise to their irregular size and shape. Their formation is associated with a transient 5- to 10-fold increase in cellular fluid uptake (101, 114). The cellular components required for macropinocytosis are intricate. While it is independent of clathrin, macropinocytosis necessitates the presence of actin, Rac family small GTPase 1 (RAC1), and Phosphoinositide 3-kinase (PI3K) (111). The verification of fluid-phase uptake via macropinocytosis presents challenges due to the non-specific nature of its cargo and the absence of signature marker proteins. However, the uptake of large molecules currently serves as the most reliable indicator of macropinocytosis. Tracer molecules exceeding 70 kDa are predominantly internalized by macropinocytosis, given that the small size of micropinosomes or endosomes restricts both the magnitude and quantity of high molecular weight molecules (111, 115). After formation, macropinosomes move deeper into the cytoplasm, where they can undergo acidification, as well as homo- and heterotypic fusion events. Depending on the cell type, they either recycle back to the cell surface or feed into the endosome network and mature before fusing with lysosomes (101, 114).

Macropinocytosis is not a universal cellular process. In immature DCs, macrophages, and podocytes, it is constitutively active (116, 117), whereas in other cells, macropinocytosis needs to be activated by extracellular stimuli, such as EGF (118). Macropinocytosis facilitates the efficient internalization of extracellular fluids, with the larger size of the vesicles also aiding in the uptake of large molecules (111). The role of macropinocytosis also varies between cell types. In immature DCs, it is employed to survey the surrounding environment for foreign pathogens (117). In *Dictyostelium discoideum* (amoeba) and Ras-transformed human pancreatic cancer cells, macropinocytosis serves as a primary route for uptake of nutrients, including albumin (119, 120). In podocytes, macropinocytosis is proposed to be essential for maintaining the function of glomerular basement membrane and filtration of the kidney by removing proteins that cross the glomerular basement membrane (111).

### 4.2 Macropinocytosis serves as a strategy of nutrient uptake in trophoblasts

Amino acids serve as a crucial input for mechanistic target of rapamycin complex 1 (mTORC1) activation in STB, and in turn, mTORC1 signaling is indispensable for amino acid uptake (121). Studies have demonstrated the importance of placental mTOR activation to facilitate plasma membrane expression of specific system A (SNAT2, SLC38A2) and system L (LAT1, SLC7A5) transporter isoforms (122, 123). Furthermore, recent research indicates that macropinocytosis may also occur in placental STB (6). During brief periods of nutrient deprivation, the inactivation of mTORC1 triggers the formation of autophagosomes to engulf intracellular components, including proteins, thereby recycling amino acids for adaptive protein synthesis and ensuring cell survival (124); while during prolonged nutrient deprivation, the persistent suppression of mTORC1 activation of Ras signaling allows the STB to utilize pinocytotic extracellular proteins as an amino acid source, addressing the increased biomass demand for sustained cell viability (124). In primary human trophoblasts and in the BeWo cell line, differentiation towards a syncytium triggers macropinocytosis, which is significantly enhanced during amino acid shortage, as induced by inhibiting mTOR signaling (6). Furthermore, inhibiting mTOR in pregnant mice significantly stimulates macropinocytosis in the STB of the placenta, whilst blocking macropinocytosis worsens the fetal growth restriction caused by mTOR-inhibition (6). Amino acids prompt the recruitment of mTORC1 to lysosomal membranes. Subsequent activation of mTORC1 by growth factor signaling enables lysosomal mTORC1 to monitor the recovery of amino acids from proteins that have been delivered via endocytosis or autophagy (124). Within the distinctive microenvironment of the placental STB, where nutrient supply is entirely reliant on maternal blood perfusion into the intervillous space, sensing of nutrient status in placental syncytium through mTOR activity may hence serve to balance transporter-mediated amino acid uptake and extracellular protein macropinocytosis (6). This mechanism offers a flexible strategy, enabling the placenta to adapt efficiently to nutrient fluctuations whilst consistently providing biosynthetic substrates for the growing fetus.

A significant unresolved issue is the fate of scavenged large molecules in STB. It is plausible that these molecules may be transported to lysosomes for degradation, subsequently serving as an energy source or recycled building blocks for cellular components, as evidenced in podocytes and tumor cells (111, 119). A deficiency in amino acids (Arg, Lys, and Glu) augments macropinocytosis in syncytialized trophoblasts, and inhibiting macropinocytosis with 5-(N-ethyl-N-isopropyl) amiloride (EIPA) has been shown to diminish fetal and placental weight, reinforcing the idea that large macropinocytosed molecules in the placental STB could potentially serve as a nutrient source for the developing conceptus (6).

### 4.3 Macropinocytosis serves as a pathway for the entry of pathogens into trophoblasts

Although typically associated with growth factor–induced fluid uptake, a variety of particles, including apoptotic bodies, necrotic cells, bacteria, viruses, and protozoans can induce dramatic, cell-wide plasma membrane ruffling. This process facilitates their macropinocytic internalization alongside fluid uptake (101). Viruses exploit macropinocytosis as a mechanism for cell entry, this includes ZIKV, SARS-CoV-2, mature vaccinia virus virions, species B human adenovirus serotype 3, echovirus 1, group B Coxsackieviruses, herpes simplex virus 1, Kaposi’s sarcoma-associated herpesvirus, and human immunodeficiency virus-1 (HIV-1) (2, 27, 101). The evolution of viruses to utilize macropinocytosis for internalization and entry may be attributed to several factors; however, the primary reason is likely particle size, especially in the case of vaccinia and herpes simplex virus 1. These viruses are presumably too large to be taken-up through most other forms of endocytosis. For other viruses, macropinocytic entry could potentially serve as a strategy to expand their host range or tissue specificity (101).

Despite the formidable nature of placental defenses, which can resist most microorganisms, select pathogens such as those from the TORCH group (Toxoplasma, Others, Rubella, Cytomegalovirus, and Herpes simplex virus) can circumvent and/or weaken these barriers for vertical transmission (125–127). The recent explosive outbreak of ZIKV, a new addition to the TORCH pathogens, has been identified as a significant threat to pregnancy, causing severe maternal and fetal outcomes including miscarriage and microcephaly. These outcomes have not previously been associated with other infectious diseases related to flaviviruses (2, 128, 129). The placenta and fetus have been found to be more susceptible to ZIKV infection during early gestation, as no apparent fetal demise or disease has been observed when ZIKV exposure occurs in the later stages of pregnancy (130, 131). This suggests that the maturation of the placental barrier over time may independently restrict fetal infection and mitigate ZIKV-related diseases (130, 131). Using gene manipulating mouse models, it was discovered that type I interferon (IFN-I) plays a crucial role in limiting ZIKV replication and transmission systemically (2). Similarly, human trophoblasts from full-term placentas have been shown to release IFNλ1 to protect the placenta from ZIKV infection through both autocrine and paracrine mechanisms (132). Despite this, several potential cell surface receptors including the TAM (TYRO3, AXL, and MER) receptor family, as well as protein S (PROS1) and growth arrest-specific gene 6 (GAS6) have been proposed to facilitate ZIKV entry into trophoblasts (133). In addition, transcytosis of ZIKV by the trophoblast cell line JEG-3 was significantly reduced by inhibitors of macropinocytosis and caveolae- and clathrin-mediated endocytosis (63). These data suggest that placental transmission of the virus may occur through multiple endocytosis pathways.

Several studies have confirmed the transplacental transmission of SARS-CoV-2 during pregnancy through the use of immunohistochemistry and *in situ* hybridization techniques to detect viral antigens or viral nucleic acid within fetal cells of the placenta (24, 134–140). However, this form of virus transmission is rare (141, 142). Despite this, patients afflicted with SARS-CoV-2 infection display cellular and molecular markers indicative of placenta insufficiency, coupled with a significant antiviral and pro-inflammatory response at the maternal-fetal interface (27). Histological observations including maternal vascular mal-perfusion, fetal vascular mal-perfusion, chronic histiocytic intervillositis, or increased intervillous fibrin have been identified in a limited number of placentas from infected women (27). As previously stated, SARS-CoV-2 primarily enters the human placenta via ACE2 and TMPRSS2, or it is internalized via CME as a virus-ACE2 complex (59–62). Although macropinocytosis is not the primary route of entry for SARS‐CoV‐2 (143, 144), the virus can activate the signaling pathways that trigger macropinocytosis. This allows the virus to enter the cell by promoting actin‐mediated membrane ruffling and lamellipodia formation at sites of membrane perturbation. Consequently, this leads to the closure and formation of large, irregular vesicles (145–147).

The best-documented example of virus entry via macropinocytosis so far is the vaccinia virus (148). Vaccinia fetalis, a rare but frequently fatal complication of primary vaccinia virus (smallpox) vaccination during pregnancy, involves the vertical transfer of the vaccinia virus from mother to fetus (149). Using liver cells as a model, researchers have discovered that vaccinia-induced macropinocytosis exhibits several distinct characteristics. Notably, the association of mature virions with cells triggers the formation of large transient plasma membrane blebs, rather than classical lamellipodial ruffles (101). Furthermore, the membrane of vaccinia is enriched in phosphatidylserine, a phospholipid essential for the macropinocytic clearance of apoptotic debris (150, 151). This suggests that vaccinia virions stimulate a macropinocytic response in host cells by simulating apoptotic bodies (148). However, further research is required to determine whether this virus is able to enter trophoblast and the human placenta through macropinocytosis.

*Listeria monocytogenes*, a Gram-positive bacterium, is the pathogen responsible for listeriosis, a foodborne disease. Symptoms of listeriosis can range from gastroenteritis and meningitis to encephalitis, maternal-fetal infections, and septicemia (152). The diverse clinical manifestations of *Listeria monocytogenes* infection underscore its ability to penetrate tight barriers, including the placental barrier in humans (152). Once ingested, the bacterium can traverse the fetoplacental barrier in pregnant women, leading to outcomes such as early pregnancy loss, stillbirth, or fetal infection (153, 154). Both traditional phagocytes, like macrophages, as well as non-phagocytic cells, including epithelial cells, endothelial cells and hepatocytes, internalize *Listeria monocytogenes* (155, 156). The entry of *Listeria monocytogenes* into non-phagocytic cells is facilitated by the bacterial surface-associated internalins A and B (InlA and InlB). InlA and InlB employ the adherens junction protein E-cadherin and the Met tyrosine kinase receptor as host ligands, and exploit the endocytic recycling machinery of these receptors, instigating cytoskeletal remodeling and facilitating bacterial internalization (157, 158). *Listeria monocytogenes* can colonize the placenta through ActA-dependent cell-to-cell spread, transitioning from infected macrophages to the STB layer, or via direct invasion of the trophoblast facilitated by InlA and InlB (159). Furthermore, InlP, a novel virulence factor for listeriosis, interacts with the host protein afadin (160), specifically enhancing *Listeria monocytogenes* transcytosis (161). The colonization of the placenta by *Listeria monocytogenes* occurs through hematogenous spread. Despite the STB being extensively exposed to maternal blood, it maintains relative resistance to *Listeria monocytogenes* infection (161). The ability of EVTs to restrict *Listeria monocytogenes* may be associated with the capacity of dNK cells to directly transfer the antimicrobial peptide granulysin to trophoblasts via nanotubes. This nanotube transfer specifically targets intracellular *Listeria monocytogenes* without damaging the trophoblast, thereby preserving the integrity of the maternal-fetal barrier (162). Other work has reported that *Listeria monocytogenes* can be taken up by human hematopoietic stem cells undergoing myeloid/monocytic differentiation through macropinocytosis (163). However, whether *Listeria monocytogenes* can be internalized by STB through macropinocytosis requires further investigation.

In addition to viruses and bacteria, protozoans can also infiltrate cells via macropinocytosis. The most well-documented example of this is *Toxoplasma gondii*, the causative agent of toxoplasmosis. This obligate intracellular protozoan parasite can invade and proliferate within any nucleated cell of a broad spectrum of homeothermic hosts (164). Predominantly, transmission occurs to the fetus in women who contract their primary infection during pregnancy. Although the majority of infants appear to be healthy at birth, significant long-term consequences can become apparent months or years later (165). The standard internalization of *Toxoplasma gondii*, often referred to as active penetration, involves several stages; initial recognition of the host cell surface, followed by sequential secretion of proteins from parasite micronemes and then rhoptries that assemble a macromolecular complex forming a specialized and transient moving junction (164, 166). The parasite is subsequently internalized through an endocytic process, leading to the formation of a parasitophorous vacuole that does not fuse with lysosomes, where the parasites persist and multiply (167, 168). Treatments that interfere with macropinocytosis, such as incubation with amiloride or IPA-3 (inhibitor targeting PAK1 activation-3), have been shown to increase parasite attachment to the host cell surface, but significantly inhibit parasite internalization when tested on various cell types (164). Immunofluorescence microscopy has revealed that markers of macropinocytosis, such as the Rab5 effector rabankyrin 5 and Pak1, are associated with parasite-containing cytoplasmic vacuoles, suggesting that macropinocytosis plays a role in the entry of *Toxoplasma gondii* into host cells (164). Beyond *Toxoplasma gondii*, other like *Leishmania Mexicana* (169), *Leishmania donovani* (170), and *Trypanosoma cruzi* (171, 172), along with certain bacteria, including *Pseudomonas aeruginosa* (173), may also enter host cells via phagocytosis. Therefore, macropinocytosis might be more frequent than currently appreciated (174). However, further research is required to determine whether parasites can gain entry into trophoblast through macropinocytosis.

## 5 *In vivo* evidence underscores the pivotal role of endocytosis at the maternal-fetal interface

The current understanding of the role of endocytosis in pregnancy maintenance and pathogen defense is largely derived from cell culture models. However, *in vivo* studies are necessary to extrapolate these findings into a physiological context. Previous research has demonstrated that RGS (G protein signaling)-PX1, also known as sorting nexin 13, is a member of the regulators of RGS and sorting nexin (SNX) protein families that can inhibit Gs-mediated signaling through its RGS domain, thereby regulating endocytosis and EGFR degradation (175, 176). In mice, genetic deficiency of Snx13 has been shown to result in severe fetal growth retardation, neural tube closure defects, blood vessel formation defects and placental labyrinth layer formation defects (177). Whole body Snx13-null embryos also showed embryonic lethality around mid-gestation (177). The visceral yolk sac endoderm cells of Snx13-null conceptuses also showed significant changes in the organization of endocytic compartments, including abnormal localization of several endocytic markers such as megalin, ARH (autosomal recessive hypercholesterolemia), LAMP2 (lysosome-associated membrane glycoprotein 2) and LC3 (microtubule-associated protein light chain 3) (177). These results suggest that Snx13-null embryos have impaired nutrient uptake and transport, which may contribute to the developmental defects and lethality observed (177).

Although direct *in vivo* evidence of the role of endocytosis in pregnancy outcomes is scarce, there is a wealth of indirect evidence. The cystic fibrosis transmembrane conductance regulator (CFTR) is an example of such a gene. It encodes a cAMP-regulated chloride channel and its mutation is the primary cause of cystic fibrosis (178). CFTR is expressed ubiquitously throughout the female reproductive tract including the cervix, ovary, oviduct and uterus (179, 180). C*ftr* ^-^ *^/-^* mice have been shown to develop chronic inflammation and gastrointestinal disease, slower growth rates and delayed puberty onset compared with wild type mice (181). Two independently derived mouse models of cystic fibrosis were used to study female infertility associated with the disease. Both models showed reduced fertility as indicated by decreased litter number and litter size. Other reproductive phenotypes in the cystic fibrosis female mice, such as small ovarian and uterine size, irregular estrous cycles and reduced oocyte ovulation rates, were also found to contribute to the reduced fertility (182). Evidence additionally indicates that CFTR plays an important role in proximal tubular endocytosis. This has been demonstrated both in the cystic fibrosis mouse model and patients with cystic fibrosis (183, 184). For instance, mice deficient in CFTR show impaired uptake of ^125^I-β2-microglobulin, reduced cubilin receptor expression in their kidneys and increased excretion of cubilin and its low molecular weight ligand into urine. These data highlight the importance of CFTR in RME within proximal tubular cells for renal handling of low molecular weight proteins (183, 184). Whether or not CFTR deficiency affects pregnancy outcomes through impaired placental endocytosis remains to be determined.

## 6 Conclusion

**Figure 2:**
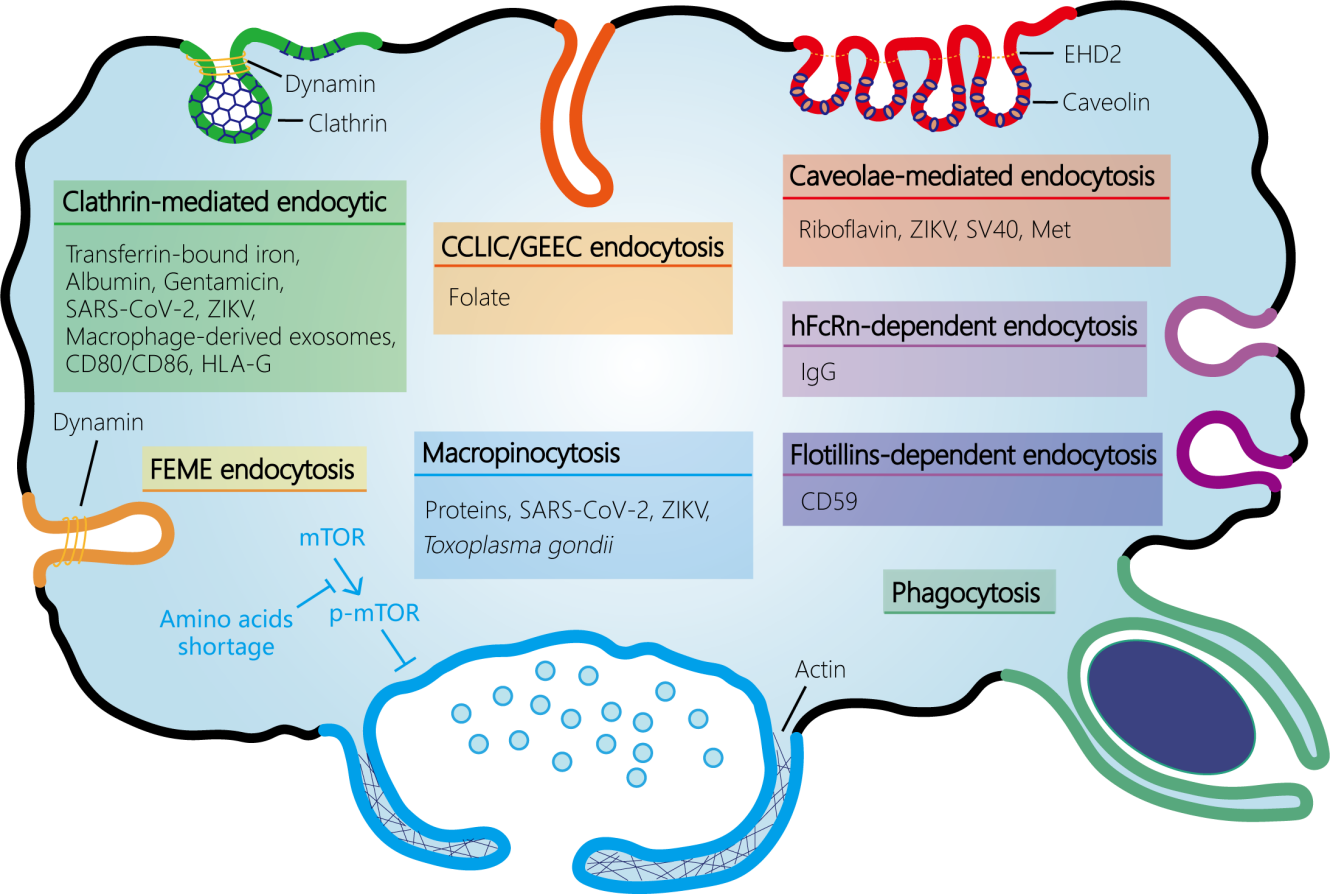
Schematic representation of the endocytosis pathways at the maternal-fetal interface. At the maternal-fetal interface, multiple endocytosis pathways exist, including receptor-mediated endocytosis (RME) and fluid-phase endocytosis. RME is subdivided into clathrin-mediated endocytosis (CME) and clathrin-independent endocytosis (CIE). CIE encompasses several types such as caveolin-mediated endocytosis, clathrin-independent carrier (CLIC)/glycosylphosphatidyl­inositol-anchored protein enriched early endocytic compartment (GEEC) endocytosis (which is independent of both clathrin and dynamin), fast endophilin-mediated endocytosis (FEME, a pathway for rapid ligand-driven endocytosis of specific membrane proteins that is independent of clathrin but dependent on dynamin), hFcRn- or flotillins-dependent endocytosis, and phagocytosis. Fluid-phase uptake in cells occurs through two separate processes: micropinocytosis and macropinocytosis, which are classified based on the size of the vesicles. Amino acids shortage activates macropinocytosis by inhibiting mTOR phosphorylation and activation in syncytiotrophoblast. SARS-CoV-2, severe acute respiratory syndrome coronavirus 2; ZIKV, Zika virus; EHD2, Eps15 homology (EH) domain-containing protein 2.

The ongoing dialogue and interaction between maternal and fetal cells are essential for the immunotolerance of the semi-allogeneic fetus, protection from pathogen infection, and nutrient transfer. These processes are mediated by various mechanisms including passive transport, facilitated diffusion, active transport, and multiple endocytosis pathways such as CME, CIE, and pinocytosis. Additionally, chemicals, drugs, and pathogens can also cross this barrier via endocytosis. Thus, endocytosis plays a critical role in successful pregnancies (**Figure 2**). Despite these findings, there remain substantial gaps in our understanding of the endocytic mechanisms that occur at the maternal-fetal interface. While research on endocytosis has advanced rapidly in other tissues, progress in human placenta has been notably slower. In addition, most of the studies have relied on the use of trophoblast cell lines rather than primary trophoblast, trophoblast organoids, or human placenta specimens. Moreover, some specific endocytosis pathways may be more common than currently thought (e.g. macropinocytosis), which has been hampered by the lack of detailed investigation into their specific pathway components and deficiency of selective inhibitors. Therefore, additional studies of endocytosis at the maternal-fetal interface are required to ensure normal fetal development through nutrient transfer and immune tolerance and to prevent pathogen infections in pregnant women and avoid adverse pregnancy outcomes.

## Author contributions

MF: Writing – original draft, Visualization. HW: Writing – original draft, Visualization. AS: Writing – review & editing. YW: Writing – review & editing, Funding acquisition. XS: Writing – review & editing, Writing – original draft, Supervision, Investigation, Funding acquisition, Conceptualization.
